# Response: Commentary: Totality of the Evidence Suggest Prenatal Cannabis Exposure Does Not Lead to Cognitive Impairments: A Systematic and Critical Review

**DOI:** 10.3389/fpsyg.2021.685328

**Published:** 2021-05-28

**Authors:** Ciara A. Torres, Christopher Medina-Kirchner, Kate Y. O'Malley, Carl L. Hart

**Affiliations:** ^1^School of Social Work, Columbia University, New York, NY, United States; ^2^Department of Psychology, Columbia University, New York, NY, United States; ^3^Division on Substance Use, Department of Psychiatry, New York State Psychiatric Institute, New York, NY, United States; ^4^Department of Psychological Sciences, Swinburne University, Hawthorn, VIC, Australia

**Keywords:** cannabis, marijuana, prenatal, cognition, normative data

We appreciate the interest Singer et al. (2021) have taken in our recent critical review of the literature assessing the impact of prenatal cannabis exposure on cognitive functioning (Torres et al., 2020a). We concluded the current evidence suggests that prenatal exposure alone is not associated with subsequent clinically significant cognitive impairments.

Singer et al. contend that nine of their papers should not have been included in our review because the “cohort was composed for a longitudinal study to assess developmental sequelae of prenatal cocaine exposure, not cannabis.” We respectfully disagree. Not only was it appropriate to include findings from the papers in our review, but it would have been less than comprehensive, and frankly, irresponsible, to exclude this information from our analysis. It is extremely troubling that the Case Western Reserve University (CWRU) group would make such an assertion, especially in light of the fact that they readily drew conclusions about the impact of prenatal marijuana exposure on cognitive functioning of offspring in their papers.

For example, Lewis et al. (2004) state that their study was “not designed to assess the effects” of prenatal marijuana exposure. Yet, in the very same paragraph, the authors claim to have found “negative effects of marijuana exposure on language” in their sample. They make this assertion, despite the fact that the marijuana-specific data consisted of a simple correlation that does not adjust for other factors including, but not limited to, substance use other than marijuana (see their Table 5). Moreover, Lewis et al. describe that their findings were consistent with two other studies (Fried and Watkinson, 1990; Day et al., 1994) both of which met criteria to be included in our critical review and were discussed in detail. Further, Lewis et al. (2010) reported no statistically significant correlations—also unadjusted for other factors—between prenatal marijuana exposure and cognition (see their Table 4). The Discussion section does not elaborate on this finding. Given the negative marijuana-related statements contained in the Discussion section of their earlier paper (Lewis et al., 2004), this is a conspicuous omission. It seems that statements about the potential role of prenatal marijuana exposure are acceptable only when deleterious marijuana-related correlations are observed.

Noland et al. (2003a,b); Noland et al. (2005) also focused on prenatal cocaine exposure. Again, this stated focus did not preclude the authors from drawing conclusions about the purported adverse connection between prenatal cannabis exposure and subsequent cognitive functioning. For instance, in the Abstract of Noland et al. (2005) it is stated, “Severity of maternal use of marijuana during pregnancy was positively correlated with omission errors, suggesting impaired sustained attention,” even though the correlation did not reach statistical significance. It would have been irresponsible for us *not* to include this type of information in a review of the literature assessing the effects of prenatal cannabis exposure on subsequent cognitive functioning of offspring. It would have been equally negligent not to include data from Noland et al. (2003a) entitled, “Executive functioning in preschool-age children prenatally exposed to alcohol, cocaine, and marijuana.” Relatedly, despite the fact that Noland et al. (2003b) found no significant associations between prenatal cannabis exposure and cognitive performance, the Discussion section of this paper directed readers to other papers that supposedly found evidence of “impair[ed] executive function in children” due to prenatal cannabis (and/or alcohol) exposure (Fried and Smith, 2001; Noland et al., 2003b). Interestingly, Noland et al. (2003b) is one of the papers used as evidence in support of this statement, even though the study did not find a relationship between prenatal cannabis exposure and cognitive functioning. The other paper, cited as evidence supporting the claim (Fried and Smith, 2001), is not even an empirical paper. It is a review of existing literature.

In general, the authors of the papers by Singer et al. were more measured in their discussions of the impact of prenatal cannabis exposure, except for one glaring departure. Singer et al. (2008) reported that the group of children exposed to cannabis prenatally performed more poorly on a measure of processing speed, as assessed by the WISC-IV. As discussed in our critical review, however, the authors did not report cognitive scores or whether the scores were within the normal range of functioning. Despite no data on the clinical importance of their findings, Singer et al. stated that their findings are “consistent with visual-cognitive deficits in adolescents” prenatally exposed to cannabis. Again, we would have been remiss to omit this information from a major review of the scientific literature assessing cognitive outcomes among individuals prenatally exposed to cannabis.

Ultimately, if a study included data on prenatal cannabis exposure and cognition, we included it in our comprehensive review of the published literature. The fact is, it would have been inappropriate to exclude such studies, including those conducted by CWRU group. Still, out of respect to these researchers, we performed a reanalysis of the data excluding the CWRU studies. The findings were nearly identical. The original analysis revealed that in 96.6% of the cognitive tests taken, prenatally-exposed children performed as well as unexposed children. The analysis without the CWRU studies revealed a difference of 0.1% (now at 96.5%). The consistency of findings further bolsters our conclusions.

Another point raised by Singer et al. (2021) is that “the Generation R Study (El Marroun et al., 2009) was inexplicably not included” in our review. It is true that we excluded these papers from our review. But the reason for this is not inexplicable, rather it's quite simple: papers from the Generation R study did not assess *cognitive functioning*. Our review focused on the empirical literature assessing the cognitive outcomes of children prenatally exposed to cannabis. Granted, one study from the Generation R cohort was initially considered for inclusion (El Marroun et al., 2011). But after careful review of the study's Methods section, we determined that it was inappropriate. The methodology consisted of asking mothers about their children's behavior (using The Child Behavior Checklist for toddlers) as a means of assessing “attention.” So we excluded this study from our analysis because a *checklist* is not a *cognitive* test.

Singer et al. (2021) also take issue with our methodology for determining clinical significance. They argue that “using a control group of similar age, race, and socioeconomic status… actually provides a more refined test of differences since it controls, either through design or statistically, confounding factors in addition to age and education.” It is true, inclusion of a control group—despite the incredible diversity of the human experience—is a keystone of the research enterprise. In the field of neuropsychology, however, a statistically significant difference between a control and experimental group does not provide enough information to determine clinical significance (i.e., functional impairments). Relatedly, Singer et al. assert that the clinical significance can be ascertained via effect size alone. This is inaccurate. An effect size is a measure of the magnitude of a difference, not a measure of clinical significance. As we noted in our original paper (Torres et al., 2020a), equating statistical significance with clinical significance is a pervasive error in the prenatal cannabis-cognition literature. This oversight can lead to harmful misapprehensions because it pathologizes normal functioning (Hart et al., 2012; Torres et al., 2020a,b). As stressed in our original article, future studies might yield either different results or data that supports our conclusion.

We hope that this exchange highlights some of the biases that permeate the literature on prenatal cannabis exposure, and that it further demonstrates the need for objective appraisals of the relevant literature.

## Author Contributions

CT wrote the initial draft and revised it with intellectual input from CM-K, KO'M, and CH at each stage. All authors contributed to the article and approved the submitted version.

## Conflict of Interest

The authors declare that the research was conducted in the absence of any commercial or financial relationships that could be construed as a potential conflict of interest.
